# Predicting miRNA-Disease Association Based on Neural Inductive Matrix Completion with Graph Autoencoders and Self-Attention Mechanism

**DOI:** 10.3390/biom12010064

**Published:** 2022-01-02

**Authors:** Chen Jin, Zhuangwei Shi, Ken Lin, Han Zhang

**Affiliations:** 1 College of Computer Science, Nankai University, Tianjin 300350, China; jinchen_cs@mail.nankai.edu.cn; 2 College of Artificial Intelligence, Nankai University, Tianjin 300350, China; zwshi@mail.nankai.edu.cn (Z.S.); ken_lin@mail.nankai.edu.cn (K.L.)

**Keywords:** miRNA-disease association, inductive matrix completion, graph autoencoder, self-attention mechanism

## Abstract

Many studies have clarified that microRNAs (miRNAs) are associated with many human diseases. Therefore, it is essential to predict potential miRNA-disease associations for disease pathogenesis and treatment. Numerous machine learning and deep learning approaches have been adopted to this problem. In this paper, we propose a Neural Inductive Matrix completion-based method with Graph Autoencoders (GAE) and Self-Attention mechanism for miRNA-disease associations prediction (NIMGSA). Some of the previous works based on matrix completion ignore the importance of label propagation procedure for inferring miRNA-disease associations, while others cannot integrate matrix completion and label propagation effectively. Varying from previous studies, NIMGSA unifies inductive matrix completion and label propagation via neural network architecture, through the collaborative training of two graph autoencoders. This neural inductive matrix completion-based method is also an implementation of self-attention mechanism for miRNA-disease associations prediction. This end-to-end framework can strengthen the robustness and preciseness of both matrix completion and label propagation. Cross validations indicate that NIMGSA outperforms current miRNA-disease prediction methods. Case studies demonstrate that NIMGSA is competent in detecting potential miRNA-disease associations.

## 1. Introduction

Micro RNAs (miRNAs) are a kind of small non-coding RNAs (about 22 nucleotides) that can regulate target mRNA expression during the post-transcriptional stage, via binding to the 3′-untranslated region of target mRNAs [1,2,3]. Thus, miRNAs can influence a series of biological processes (e.g., epigenetic regulation, cell differentiation, and basal metabolism), playing key roles in plenty of human diseases. For instance, previous research [4] has confirmed that the expression of miRNA hsa-mir-21 can facilitate the proliferation of several kinds of tumor cells, such as breast neoplasms, pancreatic neoplasms, and glioblastoma neoplasms. MiRNA mir-34a can suppress neuroblastoma via promoting tumor cell apoptosis [5]. Therefore, predicting potential miRNA-disease associations is crucial for disease prevention, diagnosis, and treatment.

Potential miRNA-disease associations can be discovered by experimental approaches or computational approaches. As computational approaches, especially machine learning algorithms, are more cost-effective and time-efficient, many machine learning-based methods were proposed to predict potential miRNA-disease associations [3,6,7]. These methods can be categorized into the following types.

1. Matrix analysis-based methods. Two commonly-used matrix analysis methods for predicting associations among biological entities are manifold regularization [8] and matrix completion [9], which respectively suggest that the association matrix follows manifold constraint or low-rank constraint. Manifold regularization based methods have been widely used for link prediction among biological entities [10,11,12]. Chen et al. [13] proposed a manifold regularized subspace learning method for detecting miRNA-disease associations. Xiao et al. [14] proposed a graph regularized non-negative matrix factorization method to predict microRNA-disease associations. Matrix completion based methods have been commonly used to infer associations among biological entities [15,16,17]. Chen et al. [18] proposed an inductive matrix completion-based method for inferring miRNA-disease associations. Li et al. [19] proposed a matrix completion algorithm for miRNA-disease associations prediction. Yu et al. [20] proposed a matrix completion algorithm for low-rank subspace learning, while incorporating label propagation for miRNA-disease associations prediction. Chen et al. [21] adopted neighborhood constraint matrix completion to predict disease-related miRNAs.
2. Graph analysis-based methods. Since the dependency among biological entities can be depicted via graphs, methods based on graph algorithms, such as bipartite graph algorithms, neighborhood sampling, and random walk, have been commonly applied in the field of bioinformatics [22,23,24]. Zeng et al. [25] proposed a structural perturbation method-based model for inferring disease-related miRNAs on bipartite miRNA-disease graph. Chen et al. [26] proposed a bipartite network projection-based method for miRNA-disease associations prediction. Xuan et al. [23] adopted a weighted neighborhood sampling algorithm for predicting potential disease-associated miRNAs. Chen et al. [24] proposed a matrix decomposition and heterogeneous graph inference-based model for miRNA-disease association prediction. Since random walk is an efficient way to learn graph representation via topologial relationships of graphs, Chen et al. [22] and Xuan et al. [27] adopted the random walk algorithm to identify potential miRNA-disease associations.
3. Heterogeneous features fusion methods. Integrating multi-source features is an efficient technique for predicting associations among biological entities [7,16,28]. Peng et al. [29] integrated multiple networks to identify potential miRNA-disease associations. Liu et al. [30] predicted disease-related miRNAs on a heterogeneous network with multiple features. Xiao et al. [31] proposed an adaptive heterogeneous feature inference model for predicting potential disease-associated miRNAs. Ha et al. [32] designed a metric learning model to fuse heterogeneous features for predicting miRNA-disease associations. Yu et al. [33] proposed a multi-layer heterogeneous network embedding model to predict potential miRNA-disease associations.
4. Deep learning methods. Neural networks have been widely used for detecting potential associations among biological entities [28,33,34]. Zeng et al. [35] adopted a neural network-based model to identify potential miRNA-disease associations. Chen et al. [36] proposed a deep-belief network for inferring disease-related miRNAs. Ji et al. [37] proposed an autoencoder for detecting miRNA-disease associations. Tang et al. [38] proposed a multi-view multi-channel graph attention networks to identify potential miRNA-disease associations. Graph Neural Networks (GNN) [39] have been proposed in deep learning on graphs. Thus, there are some recent studies for predicting associations among biological entities based on GNNs [40,41,42]. Li et al. [43] implemented an inductive matrix completion algorithm based on Graph Convolutional Networks (GCN) for predicting miRNA-disease associations. Li et al. [44] adopted graph autoencoders to identify potential miRNA-disease associations.

In this paper, we propose an inductive matrix completion-based method to predict miRNA-disease associations. Varying from the previous study [18], the inductive matrix completion algorithm in our method is implemented through neural networks, so it is called neural inductive matrix completion. Li et al. [43] implemented a neural inductive matrix completion algorithm based on GCN [45]. In previous works [18,43,44], matrix completion algorithms or graph neural networks were assigned to compute the representations of miRNAs and diseases, then the prediction scores of miRNA-disease pairs were obtained through the dot product of miRNA representations and disease representations. Hence, these works ignored the label propagation procedure for inferring potential miRNA-disease associations. Yu et al. [20] proposed a matrix completion algorithm for low-rank subspace learning and assigned label propagation for miRNA-disease associations prediction. However, in [20], matrix completion and label propagation are separate procedures, leading to decrease their effectiveness. To address this issue, our method propose an end-to-end framework by graph autoencoders to integrate matrix completion and label propagation. Graph autoencoders on miRNA graph and disease graph are competent to reconstruct score matrix through initial association matrix, which is equivalent to propagating labels on graphs. Meanwhile, graph autoencoders on miRNA graph and disease graph are capable of low-rank representation learning from miRNA space and disease space, respectively. The two graph autoencoders are trained collaboratively via neural inductive matrix completion. Since Geng et al. [46] suggested that the attention mechanism is linked to matrix factorization, we illuminate that our inductive matrix completion-based method is an implementation of self-attention mechanism. In a word, our method implement a Neural Inductive Matrix completion-based method with Graph Autoencoders (GAE) and Self-Attention mechanism for miRNA-disease associations prediction. Our method, named NIMGSA, has the following advantages.

1. NIMGSA implements inductive matrix completion through graph autoencoders, which not only ensures the low-rank property of representations from both miRNA space and disease space, but also depicts label propagation procedure through the reconstruction of association matrix.
2. NIMGSA integrates inductive matrix completion and label propagation through an end-to-end deep learning framework, which enhances the robustness and preciseness of both integrated procedures.
3. NIMGSA implements self-attention mechanism through inductive matrix completion on two graph autoencoders, which provides theoretical analysis and biological application to enhance the performance of attention-based neural networks.
4. The inductive matrix completion procedure is equivalent to training two Graph Autoencoders (i.e., GAE on miRNA graph and GAE on disease graph) collaboratively, which improves the capability for representation learning of these two GAEs.

Experiments demonstrate that NIMGSA is superior to the current state-of-the-art methods. Ablation studies demonstrate the superiority of our proposed architecture of networks. Case studies on several diseases demonstrate the capability of NIMGSA to detect new miRNA-disease associations. The source code of our model is available at https://github.com/zhanglabNKU/NIMGSA (accessed on 13 December 2021).

## 2. Materials and Methods

### 2.1. Problem Formulation

Let *m* and *n* denote the number of miRNAs and diseases, respectively, and $Y\in R^{m\times n}$ denotes the miRNA-disease association matrix. Y(i,j)=1 if miRNA *i* has been known to be associated with disease *j*, otherwise Y(i,j)=0. An algorithm predicating miRNA-disease associations requires matrix *Y*, along with miRNA similarity matrix $S_{m}\in R^{m\times m}$ (see Section 2.2) and disease similarity matrix $S_{d}\in R^{n\times n}$ (see Section 2.3), then ensures an optimal score matrix $F\in R^{m\times n}$, where F(i,j)∈[0,1] denotes the predicted score of the association between miRNA *i* and disease *j*. A higher score stands for a higher probability if miRNA *i* is associated with disease *j*.

In this paper, the dataset is retrieved from the HMDD v2.0 database (http://www.cuilab.cn/static/hmdd3/data/hmdd2.zip (accessed on 13 December 2021)) [47], including 5430 miRNA-disease associations among 495 miRNAs and 383 diseases.

### 2.2. MiRNA Similarity Matrix

Wang et al. [48] proposed a method to infer miRNA functional similarities from miRNA-related diseases. The similarity data can be downloaded from MISIM database (http://www.cuilab.cn/files/images/cuilab/misim.zip (accessed on 13 December 2021)). However, there are some miRNAs that are not included in this database. So we compute Gaussian kernel similarity for those not included miRNAs. The gaussian kernel similarity of miRNA pair (i,j) is defined as:

(1) $$GMS\left(i,j\right)=\exp(-∥Y\left(i,:\right)-Y\left(j,:\right){∥}^{2}/\theta_{m}),$$

where Y(i,:) denotes the *i*-th row of *Y*, and

(2) $$\theta_{m}=\frac{1}{m}\sum_{i=1}^{m}{∥Y\left(i,:\right)∥}^{2},$$

denotes the kernel bandwidth. In summary, miRNA similarities can be computed through:

(3) $$S_{m}\left(i,j\right)=\left\{\begin{array}{cc} MS(i,j) & \mathrm{if}\;(i,j)\;\mathrm{has}\;\mathrm{functional}\;\mathrm{similarity}\\ GMS(i,j) & \mathrm{otherwise} \end{array}\right.,$$

where MS(i,j) denotes the functional similarity of miRNA pair (i,j) from the MISIM database.

### 2.3. Disease Similarity Matrix

Wang et al. [48] and Xuan et al. [23] proposed two different models to compute disease semantic similarities. The Medical Subject Headings (MeSH) database (https://www.ncbi.nlm.nih.gov/ (accessed on 13 December 2021)) is commonly used for describing relationships among human diseases, and both of the models above are based on MeSH descriptors. At the beginning, a hierarchical Directed Acyclic Graph (DAG) is constructed from MeSH, where each node in this DAG denotes a disease, and each directed edge i→j denotes a link from disease *i* to disease *j*.

Wang et al. [48] suggested that the semantic contribution of disease pair (i,j) is computed through:

(4) $$SC_{1}\left(i,j\right)=\left\{\begin{array}{cc} 1 & j=i\\ \max\{\delta \cdot SC_{1}\left(i,t\right)|t\in \mathrm{children}\;\mathrm{of}\;j\} & j\neq i \end{array}\right.,$$

where δ is a hyperparameter and is set as 0.5 in [48]. Suppose N(i) denotes a node set including node *i* itself and its ancestor nodes in disease DAG, disease semantic similarity of disease pair (i,j) is computed as:

(5) $$DS_{1}=\frac{\sum_{t\in N(i)\cap N(j)}\left(SC_{1}\left(i,t\right)+SC_{1}\left(j,t\right)\right)}{\sum_{t\in N(i)}SC_{1}\left(i,t\right)+\sum_{t\in N(j)}SC_{1}\left(j,t\right)}.$$

Xuan et al. [23] suggested that the semantic contribution of disease pair (i,j) is computed through:

(6) $$SC_{2}\left(i,j\right)=-\log\left(\frac{\mathrm{the}\;\mathrm{number}\;\mathrm{of}\;\mathrm{DAGs}\;\mathrm{including}\;j}{\mathrm{the}\;\mathrm{number}\;\mathrm{of}\;\mathrm{diseases}}\right).$$

Then, disease semantic similarity of disease pair (i,j) is computed as:

(7) $$DS_{2}=\frac{\sum_{t\in N(i)\cap N(j)}\left(SC_{2}\left(i,t\right)+SC_{2}\left(j,t\right)\right)}{\sum_{t\in N(i)}SC_{2}\left(i,t\right)+\sum_{t\in N(j)}SC_{2}\left(j,t\right)}.$$

However, there are some diseases that are not included in the MeSH database. So we compute Gaussian kernel similarity for those unincluded diseases. The Gaussian kernel similarity of disease pair (i,j) is defined as:

(8) $$GDS\left(i,j\right)=\exp(-∥Y\left(:,i\right)-Y\left(:,j\right){∥}^{2}/\theta_{d}),$$

where Y(:,j) denotes the *j*-th column of *Y*, and:

(9) $$\theta_{d}=\frac{1}{n}\sum_{j=1}^{n}{∥Y\left(:,j\right)∥}^{2},$$

denotes the kernel bandwidth. In summary, disease similarities can be computed through:

(10) $$S_{d}\left(i,j\right)=\left\{\begin{array}{cc} DS(i,j) & \mathrm{if}\;(i,j)\;\mathrm{has}\;\mathrm{semantic}\;\mathrm{similarity}\\ GDS(i,j) & \mathrm{otherwise} \end{array}\right.,$$

where

(11) $$DS\left(i,j\right)=\frac{DS_{1}\left(i,j\right)+DS_{2}\left(i,j\right)}{2},$$

denotes the semantic similarity of disease pair (i,j). $DS_{1}$ and $DS_{2}$ is from Equations (5) and (7).

**Figure 1:**
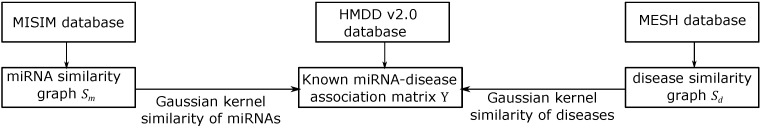
Flowchart of similarity computation.

The procedure of similarity computation is summarized as Figure 1.

### 2.4. Related Works

#### 2.4.1. Label Propagation

Previous research [49] demonstrates that label propagation is equivalent to solving manifold regularization problem [8] through fixed-point iteration. Manifold regularization assumpts that samples are distributed on a manifold, samples with higher feature similarities are closer on the manifold, and tend to share the same labels. The manifold of data can be depicted by graph structure constructed through feature matrix, which leads to graph semi-supervised learning. This type of method for biological association prediction first computes adjacency matrix from biological features to construct a graph, then propagate labels from labeled biological entities to unlabeled ones on this graph iteratively.

Suppose *L* denotes normalized Laplacian matrix of the graph, minimizing $\mathrm{trace}(F^{T}LF)$ can obtain the label matrix *F* following manifold assumption. Belkin et al. [8] added this manifold constraint to least square problem, then derived Laplacian Regularized Least Square (LRLS) method:

(12) $$\min_{F}\;\;{∥F-Y∥}_{F}^{2}+\eta \mathrm{trace}\left(F^{T}LF\right),$$

where ${∥\cdot ∥}_{F}$ denotes the Frobenius norm of a matrix, and η is a hyperparameter. Equation (12) is a trade-off between the accuracy based on labeled data, and the smoothness of the manifold. This is classified as manifold regularization [8]. Label propagation follows the framework of manifold regularization as Equation (12). Xia et al. [10] derived that association matrix *F* follows manifold assumption, and can be obtained via solving Equation (12).

Numerous research [50,51,52] demonstrate that Graph Neural Networks (GNN) is closely linked to label propagation algorithm. The outputs of GNN follow the manifold constraint. Hence, a graph autoencoder with *Y* as input and *F* as output can obtain the optimal solution of Equation (12). Simulating the label propagation algorithm through the reconstruction procedure of graph autoencoder, has been validated as an efficient way for biological association prediction in previous research [41,42].

#### 2.4.2. Inductive Matrix Completion

Natarajan et al. [15,18] proposed inductive matrix completion to predict associations among biological entities. The matrix completion problem is to approximate the initial miRNA-disease association matrix *Y* through a low-rank matrix $Z\in R^{m\times n}$. If rank(Z)≤r≤min(m,n), *Z* can be factorized into matrix $M\in R^{m\times r}$ and $D\in R^{n\times r}$, i.e., $Z=MD^{T}$. Inductive matrix completion is to optimize:

(13) $$\min_{M,D}\frac{1}{2}∥Y-S_{m}MD^{T}S_{d}^{T}{∥}_{F}^{2}+{\lambda ∥M∥}_{F}^{2}+\lambda {∥D∥}_{F}^{2},$$

where $S_{m}\in R^{m\times m}$ and $S_{d}\in R^{n\times n}$ denote similarity matrices of miRNAs and diseases, respectively. Equation (13) can be solved through a non-negative matrix factorization algorithm [53]. After obtaining optimal *M* and *D*, score matrix *F* is computed through:

(14) $$F=S_{m}MD^{T}S_{d}^{T}.$$

#### 2.4.3. Attention Mechanism

Attention mechanism [54] in the deep learning model is similar to that in cognitive science, which first calculates a probability distribution over the elements in the inputs, then takes the attention score based on this probability distribution while generating outputs.

The self-attention mechanism [55,56] is a commonly used implementation of attention mechanism. In self-attention mechanism, the input *Y* is transformed into three matrices, the Query (*Q*), Key (*K*), and Value (*V*), by three different functions. The weight assigned to each value is calculated as the dot-product of the query with the corresponding key:

(15) $$Attention\left(Q,K,V\right)=Softmax\left(\frac{QK^{T}}{\sqrt{d}}\right)V,$$

where *d* is the dimension of the vector *K*, and T is the transpose operation. The *Q*, *K*, and *V* are obtained by three linear transformations with the same input separately:

(16) $$Q=W_{Q}Y,\;\;K=W_{K}Y,\;\;V=W_{V}Y,$$

where $W_{Q}$, $W_{K}$, and $W_{V}$ are trainable parameters.

Usually, *d* is set less than the dimension of *Y*. It is obvious that the rank of Attention(Q,K,V) is no more than *d*, i.e., the attention score matrix is low-rank. Geng et al. [46] suggested that attention mechanism is linked to matrix factorization for obtaining low-rank outputs.

### 2.5. NIMGSA

#### 2.5.1. Graph Autoencoder

Suppose $Z_{m}=S_{m}M,Z_{d}=S_{d}D$, Equation (13) can be rewritten as:

(17) $$\min_{M,D}\frac{1}{2}∥Y-Z_{m}Z_{d}^{T}{∥}_{F}^{2}+{\lambda ∥M∥}_{F}^{2}+\lambda {∥D∥}_{F}^{2},$$

where $Z_{m}$ and $Z_{d}$ denote the low-rank representations of miRNAs and diseases, respectively. Previous studies [57,58] have found that autoencoders are competent to obtain low-rank representations. Therefore, we adopt graph autoencoders [59] to obtain low-rank representations in our model, NIMGSA, that $Z_{m}$ and $Z_{d}$ are learned by 2-layer graph convolution [45] encoders, respectively. The encoder is defined as:

(18) $$\mathrm{Enc}\left(A,X\right)=\tanh(A\cdot \mathrm{ReLU}\left(AXW^{\left(0\right)}\right)W^{\left(1\right)}),$$

where *A*, *X*, and *W* denote adjacency matrix, inputs, and weights, respectively.

(19) $$Z_{m}=\mathrm{Enc}\left(A_{m},Y\right).$$

(20) $$Z_{d}=\mathrm{Enc}\left(A_{d},Y^{T}\right).$$

$A_{m}$ and $A_{d}$ denote the normalized adjacency matrices of miRNA graph and disease graph, respectively.

(21) $$A_{m}=D_{m}^{-1/2}S_{m}D_{m}^{-1/2},$$

where $D_{m}$ is the degree matrix of $S_{m}$. $D_{m}$ is a diagonal matrix that is computed via $D_{m}\left(i,i\right)=\sum_{j}S_{m}\left(i,j\right)$. Similarly,

(22) $$A_{d}=D_{d}^{-1/2}S_{d}D_{d}^{-1/2}.$$

Then, Equation (17) can be rewritten as:

(23) $$\min_{W}\frac{1}{2}∥Y-Z_{m}Z_{d}^{T}{∥}_{F}^{2}+\lambda {∥W∥}_{F}^{2}.$$

The encoder-decoder architecture [54] is widely applied for reconstructing outputs from representations. The decoder is defined as:

(24) $$\mathrm{Dec}\left(A,X\right)=\mathrm{sigmoid}(A\cdot \mathrm{ReLU}\left(AXW^{\left(2\right)}\right)W^{\left(3\right)}).$$

Score matrices $F_{m}\in R^{m\times n}$ and $F_{d}\in R^{n\times m}$ can be decoded through $Z_{m}$ and $Z_{d}$, respectively:

(25) $$F_{m}=\mathrm{Dec}\left(A_{m},Z_{m}\right).$$

(26) $$F_{d}=\mathrm{Dec}\left(A_{d},Z_{d}\right).$$

Following previous research [41], since both $F_{m}\in R^{m\times n}$ and $F_{d}\in R^{n\times m}$ are low-rank provided by autoencoders, and through the rank-sum inequality that:

(27) $$\mathrm{rank}\left(\alpha F_{m}+\left(1-\alpha \right)F_{d}^{T}\right)\leq \mathrm{rank}\left(F_{m}\right)+\mathrm{rank}\left(F_{d}^{T}\right),$$

the final result:

(28) $$F=\alpha F_{m}+\left(1-\alpha \right)F_{d}^{T}$$

is low-rank, where α∈(0,1) depicts a balance between miRNA space and disease space.

#### 2.5.2. Self-Attention

In NIMGSA, $Z_{m}\in R^{m\times d}$ and $Z_{d}\in R^{n\times d}$ are equivalent to the Query *Q* and Key *K* in self-attention mechanism, which can be obtained by transformations with the same input *Y*. *F* can be regarded as Value *V* of self-attention mechanism. Similar to the definition of attention Equation (15), the attention score of the association matrix can be defined as:

(29) $$T=Softmax\left(\frac{Z_{m}Z_{d}^{T}}{\sqrt{d}}\right)⊙F,$$

where *d* is the dimension of hidden vectors, ⊙ denotes element-wise product. Then, Equation (23) can be rewritten as:

(30) $$\min_{W}\frac{1}{2}{∥Y-T∥}_{F}^{2}+\lambda {∥W∥}_{F}^{2}.$$

Then, following previous research [41,42], we add reconstruction error:

(31) $$L_{r}=\alpha ∥Y-F_{m}{∥}_{F}^{2}+\left(1-\alpha \right){∥Y-F_{d}^{T}∥}_{F}^{2},$$

into Equation (30).

(32) $$\min_{W}\frac{1}{2}{∥Y-T∥}_{F}^{2}+\beta L_{r}+\lambda {∥W∥}_{F}^{2}.$$

In NIMGSA, we set $\beta =1,\lambda =10^{-7}$.

**Figure 2:**
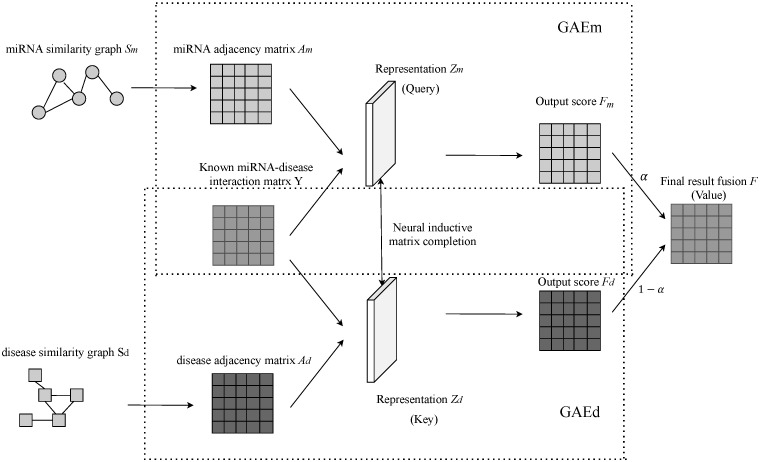
Illustration of NIMGSA. GAEm and GAEd represent graph autoencoders on the miRNA graph and disease graph respectively.

**Figure 3:**
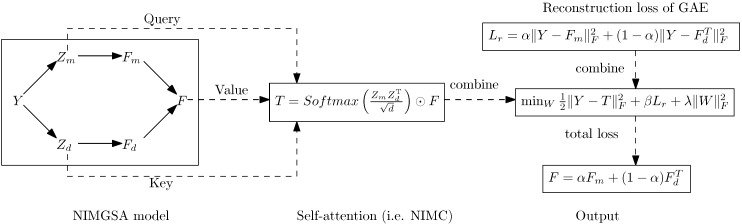
Computation procedure of NIMGSA. NIMC denotes Neural Inductive Matrix Completion.

The architecture of the NIMGSA model is illustrated as Figure 2. The procedure of NIMGSA is summarized as Figure 3 and Algorithm 1, where GAEm and GAEd represent GAEs on the miRNA graph and disease graph respectively, and NIMC denotes neural inductive matrix completion.
**Algorithm 1** NIMGSA Algorithm**Input:** 
initial association matrix *Y*, miRNA similarity matrix $S_{m}$, disease similarity matrix $S_{d}$**Output:** 
score matrix *F*1:Compute the adjacency matrix of miRNA graph $A_{m}$ and disease graph $A_{d}$ via Equations (21) and (22) respectively2:**repeat**3:   Compute Query: $Z_{m}=\mathrm{Enc}\left(A_{m},Y\right)$    // Equation (19)4:   Compute Key: $Z_{d}=\mathrm{Enc}\left(A_{d},Y^{T}\right)$    // Equation (20)5:   $F_{m}=\mathrm{Dec}\left(A_{m},Z_{m}\right)$    // Equation (25)6:   $F_{d}=\mathrm{Dec}\left(A_{m},Z_{d}\right)$    // Equation (26)7:   Compute Value: $F=\alpha F_{m}+\left(1-\alpha \right)F_{d}^{T}$    // Equation (28)8:   Compute attention score as Equation (29)9:   Train GAEm and GAEd through optimizing Equation (32)    // Update *W* (i.e., parameters of neural networks) in Equation (32) by Adam optimizer10:**until** Convergence11:**return** *F*

## 3. Results

### 3.1. Comparison with Other Methods

We compare our proposed method, NIMGSA, with other five state-of-the-art methods:

- IMCMDA: Chen et al. [18] proposed an inductive matrix completion-based method to predict miRNA-disease associations.
- SPM: Zeng et al. [25] proposed a structural perturbation method- based approach to predict miRNA-disease associations on bipartite miRNA-disease graph.
- NIMCGCN: Li et al. [43] implemented inductive matrix completion algorithm through graph convolutional networks for miRNA-disease associations prediction.
- MCLPLDA: Yu et al. [20] adopted matrix completion algorithm for low-rank subspace learning, while integrating label propagation for miRNA-disease associations prediction.
- GAEMDA: Li et al. [44] adopted graph autoencoders for miRNA-disease associations prediction.

We adopt PyTorch (https://pytorch.org/ (accessed on 13 December 2021)) to construct NIMGSA, and apply an Adam optimizer [60] to train the model. Then, we set the dropout rate [61] of neural networks at 0.5. Our model is trained on a single NVIDIA GeForce GTX 2070 GPU with 8GB of memory.

We adopt five-fold cross validation to evaluate the performance, and the metrics are listed below:

(33) $$\mathrm{Sensitivity}\left(\mathrm{SEN}\right)=\frac{\mathrm{TP}}{\mathrm{TP}+\mathrm{FN}}=\mathrm{TPR}=\mathrm{Recall}$$

(34) $$\mathrm{Specificity}\left(\mathrm{SPEC}\right)=\frac{\mathrm{TN}}{\mathrm{TN}+\mathrm{FP}}=1-\mathrm{FPR}$$

(35) $$\mathrm{Accuracy}\left(\mathrm{ACC}\right)=\frac{\mathrm{TN}+\mathrm{TP}}{\mathrm{TN}+\mathrm{TP}+\mathrm{FN}+\mathrm{FP}}$$

(36) $$\mathrm{Precision}\left(\mathrm{PRE}\right)=\frac{\mathrm{TP}}{\mathrm{TP}+\mathrm{FP}}$$

(37) $$F1\text{-}\mathrm{Score}=\frac{2\times \mathrm{Precision}\times \mathrm{Recall}}{\mathrm{Precision}+\mathrm{Recall}}$$

(38) $$\mathrm{MCC}=\frac{\mathrm{TP}\times \mathrm{TN}-\mathrm{FP}\times \mathrm{FN}}{\sqrt{\left(\mathrm{TP}+\mathrm{FN})\times (\mathrm{TP}+\mathrm{FP})\times (\mathrm{TN}+\mathrm{FN})\times (\mathrm{TN}+\mathrm{FP}\right)}}$$

where TP denotes true positive, FN denotes false negative, TN denotes true negative, FP denotes false negative, TPR denotes true positive rate, FPR denotes false positive rate, and Mcc denotes Matthews correlation coefficient. The Receiver Operating Characteristic (ROC) curve can be plotted by TPR and FPR, while the Area Under ROC curve (AUROC) and the Area under Precision-Recall curve (AUPR) are important metrics to measure the performance of a binary classification model.

**Figure 4:**
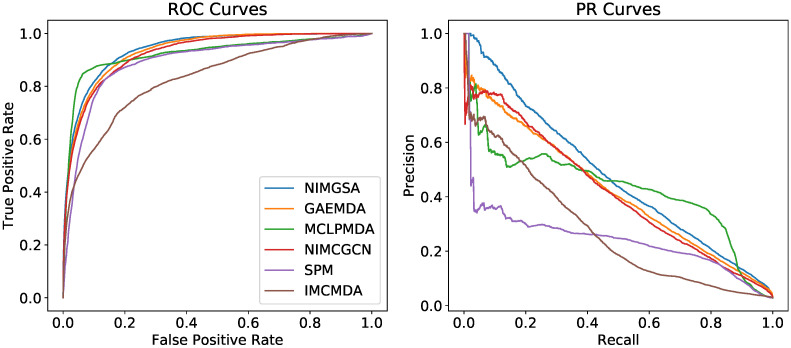
ROC and PR curves of different methods.

We plot the ROC curves and PR curves on Figure 4. The mean values and standard deviations of AUROC and AUPR are listed on Table 1. The results show that VGAELDA outperforms the other state-of-the-art methods in both AUROC and AUPR. In AUROC, NIMGSA achieves an AUROC of 0.9354, which is 0.2% higher than GAEMDA (0.9332), 0.6% higher than MCLPMDA (0.9292), 0.8% higher than NIMCGCN (0.9279), 4.3% higher than SPM (0.8960), and 11.2% higher than IMCMDA (0.8329). In AUPR, NIMGSA achieves an AUPR of 0.4567, which is 4.1% higher than MCLPMDA (0.4387), 10.2% higher than GAEMDA (0.4142), 15.8% higher than NIMCGCN (0.3943), 63.9% higher than IMCMDA (0.2785), and 85.3% higher than SPM (0.2464).

To further evaluate the performance of NIMGSA, we test our model at a high stringency level of specificity according to Equation (34). We fix specificity at 0.99, and then compute sensitivity, accuracy, precision, F1-score, and Mcc. The results are listed on Table 2, which illustrate that NIMGSA outperforms other methods at all five metrics. Matthews correlation coefficient (Mcc) is a comprehensive metric in binary classification on imbalanced data [41]. NIMGSA achieves an Mcc of 0.4273, which is higher than GAEMDA (0.4213), MCLPMDA (0.4138), NIMCGCN (0.3645), IMCMDA (0.3239), and SPM (0.2048).

### 3.2. Hyperparameter Tuning

In NIMGSA, hyperparameter α∈(0,1) depicts a balance between miRNA space and disease space. After evaluating our model at each α∈{0.1,0.3,0.5,0.7,0.9}, we find that NIMGSA performs best at α=0.5. The results are shown on Table 3.

Besides, we evaluate our model at a different learning rate in {0.001,0.01,0.05,0.1}, and the results are shown on Table 4. The results show that the best value of learning rate is 0.01.

Moreover, we evaluate our model at different dimension *d* of hidden vectors, and the results are shown on Table 5. The results depict that the performance of NIMGSA is enhanced with the increase of hidden vector dimension. However, when the dimension is larger than 64, there is little increment and the performance remains stable. Therefore, we set the hidden vector dimension at 64 to save the time and space costs.

### 3.3. Ablation Studies

To evaluate whether the components in our proposed model are necessary, we conduct ablation studies by removing individual component in our model. As shown in Equation (32), the total loss of NIMGSA consists of two parts: The self-attention loss (i.e., matrix completion loss) ${∥Y-T∥}_{F}^{2}$, and the reconstruction loss $L_{r}$. Hence, we evaluate NIMGSA with the following models.

- Self-attention: Only use self-attention loss to train the model;
- Without self-attention: Only use reconstruction loss to train the model.

As seen from Table 6, NIMGSA achieves an AUROC of 0.9354, which is 3.4% higher than the model with a self-attention loss only (0.9332), and 4.9% higher than the model without a self-attention loss (0.8916). NIMGSA achieves an AUPR of 0.4567, which is 21.2% higher than the model with a self-attention loss only (0.3768), and 34.6% higher than the model without self-attention loss (0.3392). In summary, both the self-attention loss (i.e., matrix completion loss) ${∥Y-T∥}_{F}^{2}$, and the reconstruction loss $L_{r}$, are essential for NIMGSA. Therefore, NIMGSA is a powerful model combining neural inductive matrix completion, graph autoencoders, and self-attention mechanism, to enhance the preciseness, robustness, and generalization of miRNA-disease associations prediction.

### 3.4. Case Studies

Case studies are conducted to validate the capability of NIMGSA to predict unknown miRNA-disease associations. The associations in our benchmark dataset are obtained in HMDD v2.0. We adopt three other databases to confirm the predicted miRNA-disease associations which are not included in HMDD v2.0. These three databases are dbDEMC v2.0 [62] (http://www.picb.ac.cn/dbDEMC (accessed on 13 December 2021)), miR2Disease [63] (http://www.mir2disease.org/ (accessed on 13 December 2021)), and HMDD v3.0 [64] (http://www.cuilab.cn/hmdd (accessed on 13 December 2021)). We listed the predictions of potential lncRNA-disease associations with respect to all diseases in Supplementary Table S1. The unknown disease-related lncRNAs of a disease are ranked by their predicted scores. In this paper, we adopt case studies on miRNAs associated with esophageal neoplasms, breast neoplasms, and lung neoplasms.

Esophageal neoplasms (i.e., esophageal cancer) is a major malignant cancer in digestive system [65]. NIMGSA is applied to predict potential miRNAs related to esophageal neoplasms. Supplementary Table S2 lists the top 50 predicted miRNAs associated with esophageal neoplasms. All top 50 predicted miRNAs associated with esophageal neoplasms have been confirmed. Table 7 lists the top 10 predicted miRNAs associated with esophageal neoplasms. For instance, miRNA hsa-mir-125b prevents the progression of esophageal squamous cell carcinoma through the p38-MAPK signaling pathway [66]. MiRNA hsa-mir-17 and hsa-mir-18a are prognostic indicators in esophageal squamous cell carcinoma [67]. MiRNA hsa-miR-16 induces the suppression of cell apoptosis while promoting proliferation in esophageal squamous cell carcinoma [68].

Breast neoplasms (i.e., breast cancer) is the most commonly diagnosed cancer among females worldwide [65]. NIMGSA is applied to predict potential miRNAs related to breast neoplasms. Supplementary Table S3 lists the top 50 predicted miRNAs associated with Breast Neoplasms. A total of 49 of the top 50 predicted miRNAs associated with breast neoplasms have been confirmed. Table 8 lists the top 10 predicted miRNAs associated with breast neoplasms. For instance, miRNA hsa-mir-15b targets the 3′-untranslated region of MTSS1 (metastasis suppressor protein 1), and the low abundance of MTSS1 correlates with a poor patient prognosis of breast neoplasms [69]. MiRNA hsa-mir-192 causes breast cancer cell growth arrest [70]. MiRNA hsa-miR-106a is significantly over-expressed in the breast tumor specimens compared with those in normal controls [71].

Lung neoplasms (i.e., lung cancer) is a major malignant cancer in the respiratory system [65]. NIMGSA is applied to predict potential miRNAs related to lung neoplasms. Supplementary Table S4 lists the top 50 predicted miRNAs associated with lung neoplasms. All top 50 predicted miRNAs associated with lung neoplasms have been confirmed. Table 9 lists the top 10 predicted miRNAs associated with lung neoplasms. For instance, miRNA hsa-mir-15a inhibits metastasis and lipid metabolism by suppressing histone acetylation in lung neoplasms [72]. MiRNA hsa-mir-106b plays a tumorigenesis role in non-small cell lung cancer progression by down-regulating BTG3 expression, which may lead to a novel insight to the potential biomarker and novel therapeutic strategies for non-small cell lung cancer patients [73]. MiRNA hsa-miR-16 regulates proliferation and invasion of lung cancer cells via the ERK/MAPK signaling pathway by targeted inhibition of MAPK kinase 1 (MEK1) [74].

## 4. Conclusions

Predicting potential miRNA-disease associations is important for understanding the pathogenesis of human diseases. Thus, it is crucial to infer candidate disease-related miRNAs for the scientific discovery of protecting human health. In this paper, we propose a neural network model, NIMGSA, which incorporates inductive matrix completion and graph autoencoders to detect potential miRNA-disease associations. Label propagation can be simulated through the reconstruction procedure of graph autoencoders. Meanwhile, neural inductive matrix completion algorithm not only adds collaborative training to label propagation, but also learns representations in miRNA space and disease space effeciently. Graph autoencoder is a powerful graph representation learning model that ensures the low-rank property of learned representations. Hence, the optimal score matrix can be obtained simply by the linear combination of reconstructed association matrices through GAE on miRNA graph and GAE on disease graph. NIMGSA implements self-attention mechanism through neural inductive matrix completion on two graph autoencoders, which provides theoretical analysis and biological application to enhance the performance of self-attention mechanism. Experiments demonstrated that NIMGSA is superior to the current miRNA-disease associations prediction methods in a series of statistical metrics, such as AUROC, AUPR, and Matthews correlation coefficient. Ablation studies indicate the superiority of our proposed architecture of networks. Case studies on three diseases (esophageal neoplasms, breast neoplasms, and lung neoplasms) indicate that NIMGSA is able to select candidate disease-related miRNAs.

Compared with existing miRNA-disease associations prediction methods, NIMGSA adopts an end-to-end neural network model to integrate inductive matrix completion, self-attention mechanism, and graph autoencoders. This data-driven end-to-end deep learning model not only improves the robustness and preciseness of predicting potential miRNA-disease associations, but also provides a general way for link prediction tasks of other biological entities.

## Figures and Tables

**Table 1:** Mean values and standard deviations of AUROC and AUPR, compared with different methods.

METHOD	AUROC	AUPR
IMCMDA	0.8329 ± 0.0011	0.2785 ± 0.0029
SPM	0.8960 ± 0.0070	0.2464 ± 0.0054
NIMCGCN	0.9279 ± 0.0006	0.3943 ± 0.0054
MCLPMDA	0.9292 ± 0.0069	0.4387 ± 0.0106
GAEMDA	0.9332 ± 0.0005	0.4142 ± 0.0034
NIMGSA	0.9354 ± 0.0047	0.4567 ± 0.0147

**Table 2:** Binary classification metrics of different methods on Dataset2. Sp denotes specificity. Sn denotes sensitivity. Acc denotes accuracy. Pre denotes precision. F1 denotes F1-score. Mcc denotes Matthews correlation coefficient.

SPEC	METHOD	SEN	ACC	PRE	F1-Score	MCC
0.99	IMCMDA	0.2628	0.9692	0.4365	0.3281	0.3239
	SPM	0.1551	0.9661	0.3137	0.2075	0.2048
	NIMCGCN	0.3039	0.9703	0.4725	0.3699	0.3645
	MCLPMDA	0.3567	0.9719	0.5127	0.4207	0.4138
	GAEMDA	0.3650	0.9721	0.5186	0.4284	0.4213
	NIMGSA	0.3718	0.9723	0.5229	0.4346	0.4273

**Table 3:** AUROC and AUPR at different α.

α	0.1	0.3	0.5	0.7	0.9
**AUROC**	0.9119	0.9289	0.9354	0.9338	0.9312
**AUPR**	0.3648	0.4255	0.4567	0.4556	0.4509

**Table 4:** AUROC and AUPR at a different learning rate.

lr	0.001	0.01	0.05	0.1
**AUROC**	0.9193	0.9354	0.7693	0.5557
**AUPR**	0.4077	0.4567	0.2791	0.0709

**Table 5:** AUROC and AUPR at a different dimension of hidden vectors.

DIMENSION	16	32	64	128
**AUROC**	0.9012	0.9228	0.9354	0.9357
**AUPR**	0.3642	0.4127	0.4567	0.4589

**Table 6:** Ablation studies.

Models	AUROC	AUPR
Self-attention	0.9046	0.3768
Without self-attention	0.8916	0.3392
NIMGSA	0.9354	0.4567

**Table 7:** Top 10 predicted miRNAs associated with esophageal neoplasms.

MiRNA NAME	EVIDENCE
hsa-mir-125b	dbDEMC v2.0; HMDD v3.0
hsa-mir-17	dbDEMC v2.0
hsa-mir-16	dbDEMC v2.0
hsa-mir-18a	dbDEMC v2.0
hsa-mir-19b	dbDEMC v2.0
hsa-mir-29a	dbDEMC v2.0
hsa-mir-222	dbDEMC v2.0
hsa-mir-1	dbDEMC v2.0
hsa-mir-29b	dbDEMC v2.0
hsa-mir-200b	dbDEMC v2.0

**Table 8:** Top 10 predicted miRNAs associated with breast neoplasms.

MiRNA NAME	EVIDENCE
hsa-mir-142	dbDEMC v2.0; HMDD v3.0
hsa-mir-15b	dbDEMC v2.0; HMDD v3.0
hsa-mir-192	dbDEMC v2.0; HMDD v3.0
hsa-mir-106a	dbDEMC v2.0; HMDD v3.0
hsa-mir-150	dbDEMC v2.0; HMDD v3.0
hsa-mir-130a	dbDEMC v2.0; HMDD v3.0
hsa-mir-30e	dbDEMC v2.0; HMDD v3.0
hsa-mir-92b	dbDEMC v2.0; HMDD v3.0
hsa-mir-192b	dbDEMC v2.0; miR2Disease; HMDD v3.0
hsa-mir-372	dbDEMC v2.0; HMDD v3.0

**Table 9:** Top 10 predicted miRNAs associated with lung neoplasms.

MiRNA NAME	EVIDENCE
hsa-mir-16	dbDEMC v2.0; miR2Disease; HMDD v3.0
hsa-mir-15a	dbDEMC v2.0; HMDD v3.0
hsa-mir-106b	dbDEMC v2.0; miR2Disease; HMDD v3.0
hsa-mir-141	dbDEMC v2.0; miR2Disease; HMDD v3.0
hsa-mir-15b	dbDEMC v2.0; HMDD v3.0
hsa-mir-122	dbDEMC v2.0; HMDD v3.0
hsa-mir-429	dbDEMC v2.0; miR2Disease; HMDD v3.0
hsa-mir-20b	dbDEMC v2.0; HMDD v3.0
hsa-mir-23b	dbDEMC v2.0; HMDD v3.0
hsa-mir-130a	dbDEMC v2.0; miR2Disease; HMDD v3.0

## Data Availability

The miRNA-disease association dataset is retrieved from the HMDD v2.0 database (http://www.cuilab.cn/static/hmdd3/data/hmdd2.zip (accessed on 13 December 2021)). The similarity data can be downloaded from the MISIM database (http://www.cuilab.cn/files/images/cuilab/misim.zip (accessed on 13 December 2021)). The Medical Subject Headings (MeSH) database (https://www.ncbi.nlm.nih.gov/ (accessed on 13 December 2021)) is commonly used for descripting relationship among human diseases. Case studies are conducted using dbDEMC 2.0 (http://www.picb.ac.cn/dbDEMC (accessed on 13 December 2021)), miR2Disease (http://www.mir2disease.org/ (accessed on 13 December 2021)), and HMDD v3.0 (http://www.cuilab.cn/hmdd (accessed on 13 December 2021)). Source code of our model is available at https://github.com/zhanglabNKU/NIMGSA (accessed on 13 December 2021).

## Supplementary Materials

The following are available online at https://www.mdpi.com/article/10.3390/biom12010064/s1, Table S1: Predictions of potential miRNA-disease association. Table S2: Top 50 predicted miRNAs associated with esophageal neoplasms. Table S3: Top 50 predicted miRNAs associated with breast neoplasms. Table S4: Top 50 predicted miRNAs associated with lung neoplasms.
